# Advanced Synthesis of Conductive Polyaniline Using Laccase as Biocatalyst

**DOI:** 10.1371/journal.pone.0164958

**Published:** 2016-10-14

**Authors:** Felipe de Salas, Isabel Pardo, Horacio J. Salavagione, Pablo Aza, Eleni Amougi, Jesper Vind, Angel T. Martínez, Susana Camarero

**Affiliations:** 1 Centro de Investigaciones Biológicas, CSIC, Ramiro de Maeztu 9, 28040, Madrid, Spain; 2 Instituto de Ciencia y Tecnología de Polímeros, CSIC, Juan de la Cierva 3, 28006, Madrid, Spain; 3 Novozymes A/S Krogshoejvej 36, 2880, Bagsvaerd, Denmark; Universidad Nacional Autónoma de México, MEXICO

## Abstract

Polyaniline is a conductive polymer with distinctive optical and electrical properties. Its enzymatic synthesis is an environmentally friendly alternative to the use of harsh oxidants and extremely acidic conditions. 7D5L, a high-redox potential laccase developed in our lab, is the biocatalyst of choice for the synthesis of green polyaniline (emeraldine salt) due to its superior ability to oxidize aniline and kinetic stability at the required polymerization conditions (pH 3 and presence of anionic surfactants) as compared with other fungal laccases. Doses as low as 7.6 nM of 7D5L catalyze the polymerization of 15 mM aniline (in 24 h, room temperature, 7% yield) in the presence of different anionic surfactants used as doping templates to provide linear and water-soluble polymers. Aniline polymerization was monitored by the increase of the polaron absorption band at 800 nm (typical for emeraldine salt). Best polymerization results were obtained with 5 mM sodium dodecylbenzenesulfonate (SDBS) as template. At fixed conditions (15 mM aniline and 5mM SDBS), polymerization rates obtained with 7D5L were 2.5-fold the rates obtained with commercial *Trametes villos*a laccase. Moreover, polyaniline yield was notably boosted to 75% by rising 7D5L amount to 0.15 μM, obtaining 1g of green polyaniline in 1L-reaction volume. The green polymer obtained with the selected system (7D5L/SDBS) holds excellent electrochemical and electro-conductive properties displayed in water-dispersible nanofibers, which is advantageous for the nanomaterial to be readily cast into uniform films for different applications.

## Introduction

Organic polymers capable of conducting electricity upon partial oxidation-reduction have attracted increasing attention in recent years to replace metals and semiconductors as conductors in storage devices, electromagnetic screens and conducting fibers. In particular, polyaniline (PANI) is attractive among the polymeric materials available due to a unique combination of easy processability and highly stable conductivity, with diverse optical and mechanical properties accessible in a wide range of nanostructures [1,2]. Conductivity/resistance can be rapidly changed by acid doping and base dedoping providing PANI with many of the properties desired for a chemical sensor [3]. In addition, PANI-based composite materials are also opening new dimensions in polymer electronics. PANI-graphene supercapacitors provide high specific capacitance and stability during recharging [4,5]. Thermal and conductive composites recently obtained from a combination of PANI and thermosensitive hydrogel might be useful to build electronic sensors of pressure and switch-controlled by temperature [6].

Polyaniline consists of lineal chains of *p*-coupled aniline units. The combination of benzenoid (amine N) and quinoid (imine N) rings leads to three different oxidation states of PANI: leucoemeraldine, emeraldine and pernigraniline (S1 Fig). The redox state of the polymer and the degree of protonation are responsible for different optical and electrical properties. The emeraldine salt (green-colored polymer) is the electro-conductive form of PANI (polaron) [7]. Aniline polymerization is conventionally achieved by chemical oxidation under harsh conditions using ammonium peroxydisulfate, potassium dichromate or ferric chloride as oxidant in highly acidic solutions and usually results in complex by-products. The formation of a polymer chain starts with the oxidation of aniline monomer. Emeraldine base is formed in the course of the growth of the *p*-coupled chain, and emeraldine salt is later obtained by protonation of the imine nitrogen atoms of emeraldine base with strong acids. This process is referred to as “doping” [8].

The enzymatic oxidation of aniline for the synthesis of conducting PANI constitutes an environmentally friendly alternative to the chemical polymerization because it is carried out under milder conditions. Even if peroxidases and laccases have been both explored as biocatalysts for aniline polymerization [9,10], laccases offer important operational advantage over peroxidases as they do not require stepwise addition of hydrogen peroxide to catalyze the reaction. Besides, peroxidases are sensitive to inactivation by hydrogen peroxide [11] whereas laccases only require oxygen from the air to oxidize the arylamine, releasing water as the only by-product.

Laccase oxidizes aniline monomers, dimers and oligomers. After that, the polymerization apparently proceeds by non-enzymatic coupling of the oxidized products [12]. The mixture of aniline polymers might be as complex as varied are the conditions used for the synthesis. Thus, a particular challenge is to control the reaction conditions to attain the desired product, avoiding over-oxidized or side-effect products. The use of templates favors the desired (linear head-to-tail) aniline polymerization over unwanted (side-chain branching) coupling reactions. Anionic surfactants serve as doping templates and also as amphiphilic systems to solubilize PANI by forming micelles or vesicles [12,13]. From a practical point of view, water soluble or dispersible conducting PANI is more promising by contrast to the poor solubility of the chemically-obtained polymer in common organic solvents [9].

In this study, we use a high-redox potential laccase developed in our laboratory (7D5L) as the biocatalyst of choice for the synthesis of green polyaniline due to its superior ability to oxidize aniline and high stability at preferred reaction conditions [14]. Different anionic surfactants are assayed as doping templates and the resulting polymers are fully characterized. Thus, we set up the conditions for the enzymatic synthesis of electro-conductive emeraldine easily processable in water with reliable conversion yields.

## Material and Methods

### Reagents

Citrate-phosphate buffer was prepared with Na_2_HPO_4_ and citric acid purchased from Merck Millipore. N,N’-dimethyl-p-phenylenediamine (DMPD), N-methyl-2-pyrrolidone (NMP), aniline, sodium dodecyl sulfate (SDS), docusate sodium salt (AOT) and sodium dodecylbenzenesulfonate (SDBS) were all from Sigma Aldrich. Sodium lauryl ether sulfate (SLES) (40%) was obtained from Gran Velada. Tetrahidrofuran (THF) and N,N-dimethylformamide (DMF) were obtained from LabsScan. 2,2'-Azinobis (3-ethylbenzothiazoline-6-sulfonic acid) diammonium salt (ABTS) was purchased from Roche. Chemical synthesized emeraldine salt (average Mw>15,000 Da) was purchased from Sigma Aldrich. All chemicals were of reagent-grade purity.

### Strains and culture media

The protease deficient *Saccharomyces cerevisiae* BJ5465 strain (LGCPromochem) transformed with the shuttle pJRoC30 vector carrying either 3A4, 7A12 or 7D5 chimeric laccases under the control of the GAL1 promoter were obtained in a previous work [14]. Transformed yeast cells were grown for 3 days, in flasks, in laccase expression medium supplemented with ethanol and copper to obtain the crude laccases used in this study [15]. PM1 laccase was produced in GAE medium [16], *Pycnoporus cinnabarinus* laccase (PcL) was provided by INRA-Marseille and *Myceliophtora thermophila* (Novozym 51003) and *Trametes villosa* (Novozym 51002) laccases (MtL and TvL, respectively) were provided by Novozymes (Denmark).

### Enzyme characterization

#### Determination of laccase activity

Laccase activity was measured with 20 μl samples and 180 μl of 3 mM ABTS (ABTS cation radical ε418 = 36000 M^-1^ cm^-1^, [17] or 5 mM DMPD (ε550 = 4134 M^-1^ cm^-1^, determined in this study) in triplicate, using a SpectramaxPlus (Molecular Devices) plate reader in kinetic mode. One activity unit (U) was defined as the amount of enzyme needed to transform 1 μmol substrate/minute at room temperature.

Laccase activity in the presence of templates was measured by adding 20 μl of enzyme diluted to 0.1 U/mL of activity (measured with ABTS) to 180 μl of 3 mM ABTS in 50 mM citrate-phosphate buffer pH 3.0 and different concentrations of SDS, SDBS, AOT or SLES (0.25–3.2 mM).

Oxidation of 300 mM aniline was followed at 410 nm (ε410 = 1167 M^-1^ cm^-1^ determined in this study) in 50 mM citrate-phosphate buffer pH 3.0.

#### Enzyme stability at pH 3

Laccase samples with 0.1 U/mL activity (measured with ABTS) were incubated in 50 mM citrate-phosphate buffer, pH 3.0 in the presence or absence of template (15 mM SDS), for 24 h, at room temperature. Laccase activity was measured at different time points with ABTS as aforementioned. Residual activities were calculated as a percentage of the initial activity.

#### Enzyme thermostability

Laccase samples with 0.1 U/mL activity (measured with ABTS) were incubated at 70°C for 10 minutes. Then, aliquots of 20 μl were chilled on ice for 10 min and incubated at room temperature for another 5 min before adding 180 μL of 3 mM ABTS in 50 mM citrate-phosphate buffer pH 3.0. Residual activities were calculated as aforementioned.

### Enzymatic polymerization of aniline

#### Evaluation of different anionic surfactants as doping templates

15 mM Aniline was polymerized with 0.1 U/mL of laccase (measured with ABTS) in aqueous medium buffered with 50 mM citrate-phosphate, pH 3.0, in the presence of SDS, SDBS, AOT or SLES in a concentration range of 0.6–15 mM. The reaction was carried out for 24 h at room temperature and constant stirring, in 12.5 mL reaction volume (Pyrex bottles), maintaining a liquid:air 1:1 v/v ratio. Samples were precipitated and washed with absolute ethanol to purify the polymer [13]. Purified and non-purified polyaniline were lyophilized in a Telstar lyophilizer.

#### Polymerization assays with pure 7D5 laccase

Assays were carried out as aforementioned with 5 mM SDBS as template and 0.1 U/mL (7.6 nM enzyme concentration), 1 U/mL (76 nM) or 2 U /mL (0.15 μM) of pure 7D5 laccase produced in *Aspergillus oryzae*. Samples were taken at different time-points and measured in triplicate by following the increase of absorbance at 800 nm typical for the emeraldine salt. The enzyme was produced in *Aspergillus oryzae* [18], in standard MDU-2BP media containing CuSO_4_ and purified by two ion-exchange and one size-exclusion chromatographic steps: i) anion-exchange chromatography using a Q-sepharose column and a 75 mL gradient of 0–100% elution buffer (20mM Tris pH 7 + 0.5M NaCl pH 7); ii) molecular exclusion chromatography using a HiLoad 16/600 Superdex 75 pg column (20 mM Tris-HCl + 150 mM NaCl, pH 7); iii) an anion-exchange chromatography using a Mono Q HR 5/5 column and a 30 mL gradient of 0–25% elution buffer (20 mM Tris-HCl + 1 M NaCl, pH 7). All columns are from GE Healthcare. Fractions containing laccase activity were pooled, dialyzed and concentrated between each chromatographic step.

#### Polymerization assays at fixed conditions

5 mM of aniline was polymerized with either 0.1 or 2 U/mL of crude enzyme (activity measured with ABTS) in the presence of 5 mM of SDBS as template, in 50 mM citrate-phosphate buffer pH 3.0. The reaction was carried out in 100 mL-flask (50 mL final volume) or 2 L flask (1L final volume), at room temperature and constant stirring for 24 h.

### Characterization of polymers

#### Matrix-assisted laser desorption/ionization-time of flight (MALDI-TOF) mass spectrometry (MS)

Lyophilized samples were resuspended in water, 10% ethanol, DMF or THF before the analysis. The best spectra were acquired with DMF as solvent. The measurements were taken in a MALDI-TOF-TOF Autoflex III from Bruker calibrated with Bruker peptide. The matrix used was 10 mg/mL 2,5-dihydroxybenzoic acid in DMF.

#### Spectroscopic analyses

UV-visible absorbance spectra of water-soluble PANI samples were acquired in a UV 1800 spectrophotometer (Shimadzu) using quartz cuvettes. Commercial emeraldine salt was diluted in 50% dimethylformamide. FTIR spectra of PANI were obtained in a Jasco FTIR-4200 spectrophotometer from KBr pellets of samples previously dried in an aeration oven.

#### Cyclic voltammetry

The electrochemical measurements were conducted using Dropsens screen printed electrodes (DRP-110) in a DropSens μStat400 potentiostat. For sample preparation, 0.01 g of PANI was dispersed in 5 mL of NMP. Then, 2 μL of each solution were drop-casted onto the carbon working electrode and dried under vacuum. The electrolyte employed was 1 M HCl.

#### Scanning electron microscopy (SEM)

Lyophilized PANI samples were metalized with an alloy of Au/Pd in an 80:20 ratio and a plasma current of 5–10 mA by a SC7640 Polaron sputter coater from Quorum Technologies (East Sussex, United Kingdom). The images were taken in a FE-SEM Hitachi model SU8000 (Tokio, Japan) with an acceleration voltage of 1.5 kV.

#### Conductivity

Direct current (DC)-conductivity measurements were carried out using the four-probe method on pellets obtained from dried purified PANI pressed into a disk. The measurements were carried out using a four-probe setup equipped with a DC current source (LCS-02) and a digital micro-voltmeter (DMV-001) from Scientific Equipment and Services. Prior to conductivity measurements, the polymers were re-doped with 1 M HCl.

#### Dynamic light scattering (DLS)

DLS experiments were carried out in a Protein Solutions DynaPro MS/X instrument (Protein Solutions, Piscataway, NJ) at 20°C using a 90° light scattering cuvette. Prior to the analysis, the samples were centrifuged during 20 min at 8,000 g and 20°C. Data were collected and analyzed with Dynamics V6 Software.

## Results and Discussion

### Selection of biocatalyst

**Fig 1:**
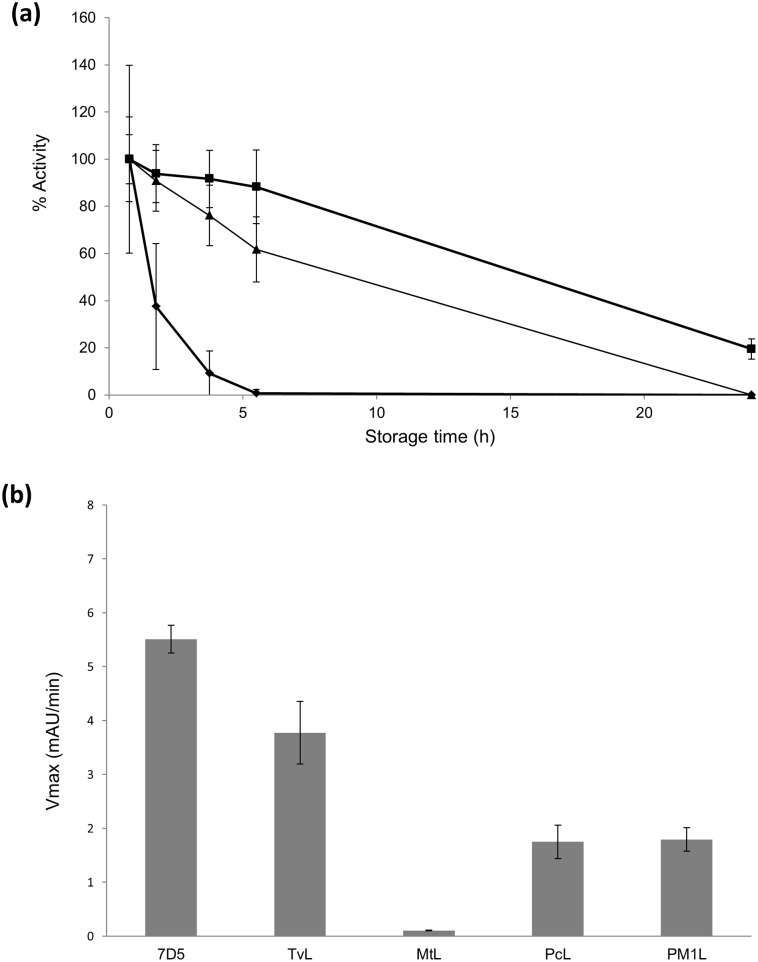
Stabilities of the engineered laccases expressed in *S*. *cerevisiae* in citrate-phosphate buffer pH 3 with 15 mM SDS, in the presence of 15 mM aniline; 7D5L (squares), 7A12L (diamonds), 3A4L (triangles) (a). Oxidation of 100 mM aniline (at pH 3.5) by 7D5L, the wild type laccases PM1L and PcL, and TvL and MtL commercial laccases (b).

From a set of high-redox potential laccases engineered and expressed in *S*. *cerevisiae* [14], three thermostable laccases, namely 3A4, 7A12 and 7D5, were evaluated at the preferred conditions for the synthesis of polyaniline (acid pH and presence of anionic surfactant). Of these, 7D5 laccase (7D5L) resulted the most stable (Fig 1a) and it was selected for further studies. Then we compared the oxidation of aniline by 7D5L and other fungal laccases such as the wild-type laccases from *P*. *cinnabarinus* (PcL), the basidiomycete PM1 (PM1L), or the commercial laccases from *M*. *thermophila* (MtL) or *T*. *villosa* (TvL) (Fig 1b). The activity of 7D5L on aniline was notably superior to the rest of the enzymes tested. Further comparison of 7D5L with TvL (the second best laccase oxidizing aniline) showed the stability of both enzymes at pH 3 (room temperature) and the higher stability of 7D5L at high temperature (70°C). Also, 7D5L has twice as high relative activity on aromatic amines (DMPD), respecting the activity with ABTS, than TvL (Table 1).

**Table 1:** Comparison of 7D5L and *T*. *villosa* laccase (TvL) for stability at acid pH and high temperature, and relative activity on aromatic amines (DMPD) respecting the activity with ABTS.

	Half-life pH 3 (h)	10 min at 70°C (%)	DMPD/ABTS activity
7D5L	22	76	1.4
TvL	20	32	0.6

The oxidation of aniline by laccase at pH 3 is hampered because at this pH aniline is mostly protonated, in the form of anilinium cation (pKa = 4.6), and the cation (E° = 1.05 V) is much less oxidizable than neutral aniline (E° = 0.63 V) [19,20]. Hence, the use of high-redox potential laccases such as 7D5L, PcL, PM1L or TvL (E° ~ + 0.8 V) is required to overcome the high potential barrier for oxidizing aniline in acidic medium [21]; whereas MtL, with a lower redox potential, is unable to catalyze the reaction. Even so, the polymerization reaction with high-redox potential laccases proceeds slowly, in several hours. The superior ability of 7D5L to oxidize aniline over other high-redox potential counterparts is likely related to its higher relative activity on aromatic amines, but its optimum pH (pH 3) for aniline oxidation (compared to pH 4.5 for TvL) and kinetic stability at the working conditions might also contribute to this enhancement. Consequently, 7D5L is the biocatalyst of choice to synthesize polyaniline in this study.

### Enzymatic polymerization of aniline

**Fig 2:**
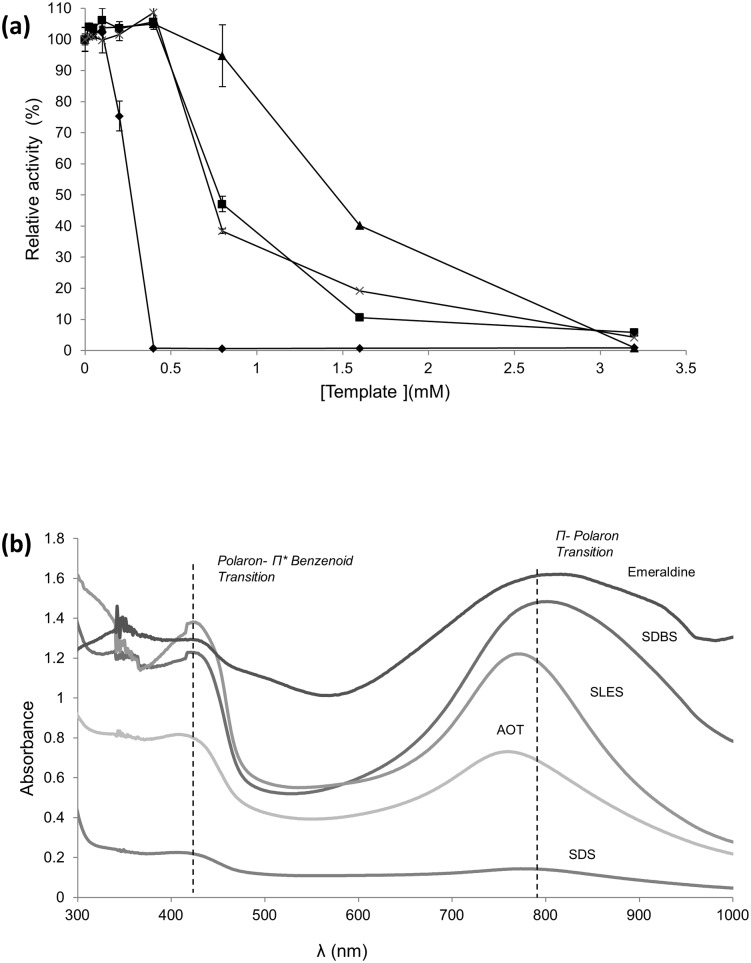
Activity of 7D5L in the presence of SDS (triangles), SLES (crosses), AOT (squares) or SDBS (diamonds) (a). UV-visible absorbance spectra of green PANI synthesized after 24 h of reaction with 7D5L (0.1 U/mL) using different anionic surfactants as templates (15 mM aniline and template were used in all cases). The spectrum of commercial emeraldine salt is provided for comparison (b).

Our final goal is the biosynthesis of water-soluble PANI with electrochemical and electro-conductive capabilities (emeraldine salt). To achieve these properties, we used 50 mM citrate-phosphate buffer pH 3, as doping agent to maintain the aniline monomer protonated. We also assayed different anionic surfactants, SDS, SDBS, AOT and SLES, to serve as i) templates to facilitate the *p*-directed coupling of the monomers, ii) as anionic dopants to get PANI in its conductive state, and iii) to make the polymer soluble in water by aggregation in micelles (S2 Fig). First, since the critical micelle concentrations of anionic surfactants are high and their negative effect on the activity of enzymes is large [22], we evaluated the activity of 7D5L in the presence of the different anionic surfactants. The residual enzyme activity was as follows: SDS >AOT> SLES> SDBS (Fig 2a). On the other hand, SDBS displays a lower critical micelle concentration (1.3 mM) than AOT (2.5 mM) or SDS (8.3 mM) in aqueous solution [23,24], which can be beneficial for the synthesis of soluble PANI at low template concentrations. By contrast to the important loss of activity observed in the absence of a reducing substrate, it is worth mentioning the "protective" effect that aniline plays on the enzyme against the presence of anionic surfactants, which allows 7D5L to be active for hours even if high concentrations of anionic surfactants are used (Fig 1a).

**Fig 3:**
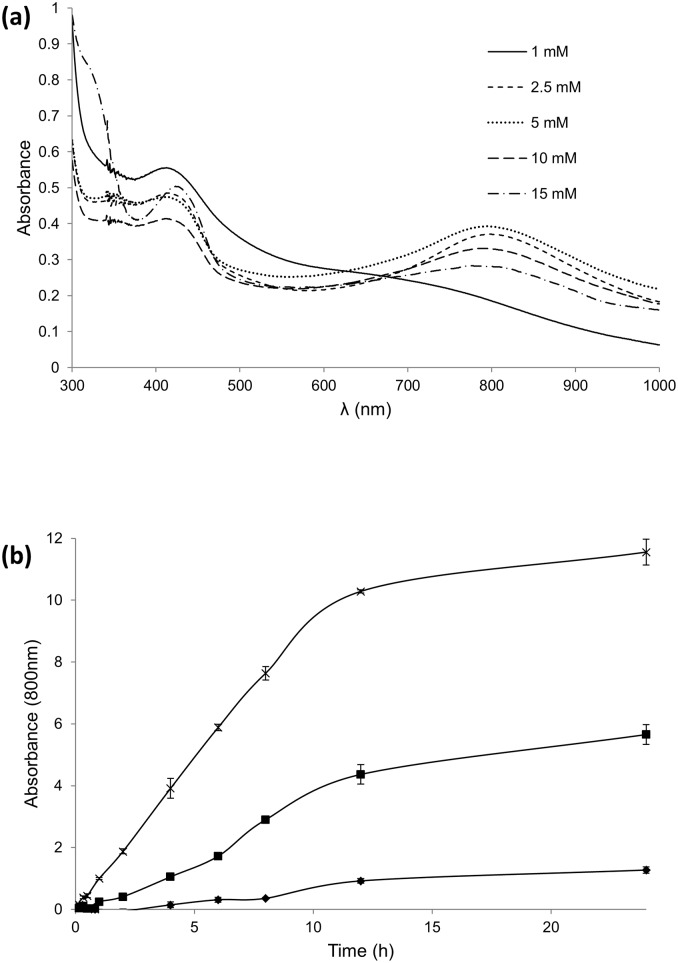
UV-visible spectra of aniline polymerized for 24 h with 0.1 U/mL of 7D5L and different concentrations of SDBS (10-fold diluted) (a). Aniline (15 mM) polymerization followed by the increase of absorbance at 800 nm, using 5 mM SDBS and 0.1 (diamonds), 1 (squares) or 2 U/mL (crosses) of pure 7D5L produced in *A*. *oryzae* (b).

After 24 h of reaction with crude laccase (0.1 U/mL), the type and amount of template relative to a fixed amount of aniline (15 mM) determined the product's properties as regards color, polymerization degree, structure and electrochemical properties. Soluble green PANI was obtained with different templates when using template/aniline ratios from 1 to 0.2, whereas a large excess of aniline respecting the template (e.g. 0.6 mM AOT) led to dark-colored precipitates due to the collapse of micelles [2]. UV-visible spectroscopy analysis of the green PANI synthesized using template/aniline molar ratio of 1 evidenced the absorption bands typical for emeraldine salt (Fig 2b) at 420 nm, characteristic of the semiquinoid radical cation [25], and 800 nm, the distinctive signal of doped PANI due to π–Polaron electronic transitions [12]. However, the magnitude of the latter varied with the different templates used as follows: SDBS>SLES>AOT>SDS. What is more, only the polymer obtained with SDBS showed the maximum at 800 nm (Fig 2b), whereas the rest showed the peak around 750 nm, suggesting partial doping [26]. Then, we decreased SDBS content (relative to 15 mM aniline) to determine the minimum amount of this template required for the synthesis of emeraldine salt. The polaron absorption at 800 nm reached its maximum with 5 mM SDBS in the reaction, whereas higher SDBS concentrations provided lower absorbance values and 1 mM of template was not sufficient to detect the polaron signal (Fig 3a). By comparison with other templates, the absorbance at 800 nm was 4-fold higher for PANI obtained with 5 mM SDBS than for PANI obtained with 5 mM AOT (data not shown).

To sum up, even though SDBS was the anionic surfactant producing the strongest loss of enzyme activity, it endowed the best performance as doping template for the enzymatic synthesis of green PANI. In addition to the aforementioned "protective" effect of aniline that increases the enzyme's tolerance to every anionic surfactant, this apparent contradiction is most likely related to the lower critical micelle concentration of SBDS respecting the other surfactants tested, thus offering better polymerization results with less template. Hence, 5 mM SDBS as template was fixed for the next polymerization assay with increasing amounts of 7D5 laccase and 15mM of aniline. The maximum polymerization rate (measured by the increase of polaron signal at 800 nm) was remarkably raised (0.37 < 6.37 < 14.64 mUA/min) in direct correlation with the amount of enzyme used (0.1 < 1 < 2 U/mL), suggesting the catalytic role of the enzyme in the polymerization (Fig 3b). In all cases, polymerization slowed down from roughly 8–12 h onwards, most probably due to the inactivation of the enzyme. It is worth mentioning that this assay was carried out with pure 7D5L produced in *Aspergillus oryzae* using enzyme concentrations between 7.6 nM (0.1 U/mL) and 0.15 μM (2 U/mL). The fact that 7D5L can be produced in industrial relevant scale by the host *A*. *oryzae*, maintaining its outstanding features for the synthesis of emeraldine, is of significance for the potential use of the biocatalyst at higher scale.

**Fig 4:**
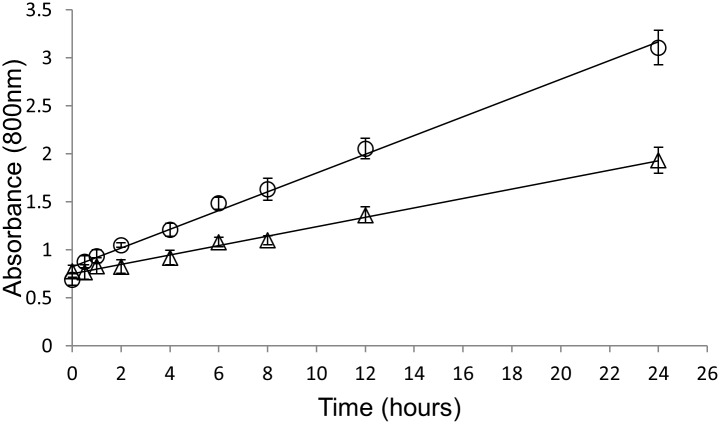
Polymerization rates (followed by the increase of absorbance at 800 nm) during 24 h of reaction with 0.1 U/mL of 7D5L and TvL using 15mM aniline and 5 mM SDBS.

Finally, we compared 7D5L and TvL for aniline polymerization at the established conditions, using 0.1 U/mL of crude enzymes. Polymerization rates were 2.5-fold higher for 7D5L (1.8 mUA/min) than for TvL (0.7 mUA/min). (Fig 4). Besides, the final yield in emeraldine salt (isolated with ethanol) obtained after 24 h of reaction with crude 7D5L was boosted from about 7% to 75% when the enzyme dose was raised from 0.1 U/mL to 2 U/mL.

### Characterization of PANI

#### Oligomers/polymers

Oligomer components were detected by MALDI-TOF-MS analysis in several of the PANI samples obtained, conforming to the co-existence of oligomers and polymers during aniline polymerization [27]. Indeed, high molecular mass polymeric fractions were not detected, most probably because of instrumental limitations. We found oligomerization degrees of up to 19 residues in the dark PANI obtained with 0.6 mM AOT and of 7–8 residues in the soluble green products obtained with 15 mM template (S3a–S3c Fig). By contrast, no oligomers were detected in the green PANI synthesized with 2.5 mM or 5 mM of SDBS as template, suggesting greater polymerization yields under these conditions (data not shown). In line with this hypothesis, oligomers were neither observed in commercial emeraldine salt (S3d Fig).

#### Emeraldine salt features revealed by FTIR

**Fig 5:**
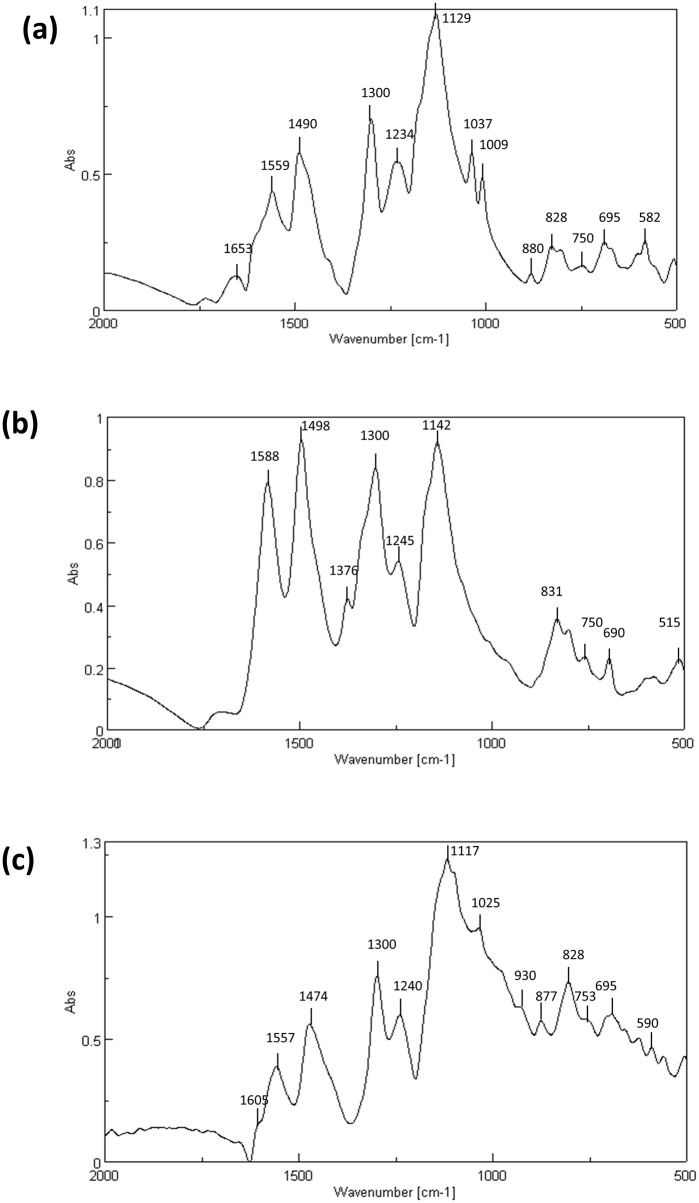
FTIR spectroscopy of PANI showing the representative bands for emeraldine salt. PANI obtained with 7D5L, 15 mM aniline and 5 mM SDBS (a). Same polymer after removal of SDBS with ethanol (b). Commercial emeraldine salt (c).

In concordance with UV-visible spectroscopy results, PANI obtained with 7D5L in the presence of SDBS clearly exhibited the characteristic FTIR bands of emeraldine salt (Fig 5a). Bands around 1560 and 1490 cm^-1^ respectively correspond to stretching vibrations of the quinoid ring (Q) and benzenoid ring (B), whereas 1300 cm^-1^ band reveals C–N stretching vibration of secondary amines in the doped form [28], and 1245 cm^-1^ band is characteristic of the conducting protonated form of PANI (assigned to C–N^●+^ stretching vibration in the polaron lattice). The ratio of the maximum intensity of the first two bands (IQ/IB) represents an estimation of the oxidation degree of polyaniline: when it approaches one, it is assumed that PANI is in the emeraldine form. In our case, the IQ/IB ratio of PANI obtained with 5 mM SDBS as template was apparently near to 1. The typical absorbance band around 1140 cm^-1^, indicating the protonation of the PANI backbone (B–NH^+^ = Q or B–NH^●+^–B vibration), was more evident after removal of template with ethanol (Fig 5b). However, the appearance of a small band around 1380 cm^-1^ suggested partial de-doping to the base form after the template's removal (Fig 5b). The sulfonate groups of the template contributed to 1130 cm^-1^ (with a characteristic band around 1180 cm^-1^) and 1037 cm^−1^ signals (Fig 5a) [29].

In the substitution region (900 to 650 cm^–1^), 828 cm^−1^ band confirmed the dominating *p*-coupled chains (due to the out-of-plane deformation of C–H in 1,4-disubstituted benzene ring and Q ring deformation), whereas bands at 753 and 695 cm^–1^ respectively correspond to the out-of-plane C–H deformation and ring bending of the monosubstituted phenylene ring. On the other hand, bands at 880 and 582 cm^–1^ can be attributed to sulfonate counterions. In particular the peak at 582 cm^−1^ assigned to SO_3_^−^ group from SDBS [29], disappeared after PANI isolation with ethanol [28,30].

The use of the other anionic surfactants rendered dissimilar FTIR spectra, with significant differences in the absorbance peaks respecting the typical signals of the emeraldine salt (Fig 5c).

#### PANI morphology

Polyaniline structure greatly depends on the external templates that direct the nano-structural growth of the polymer in or around self-assembled micelles [1]. The number of nano- and micro-scale structures for polyaniline has no paragon in other organic nano-materials [31]. Given that structure determines nanomaterial’s properties, we examined the structure and size of the polymers synthesized here by SEM and DLS.

**Fig 6:**
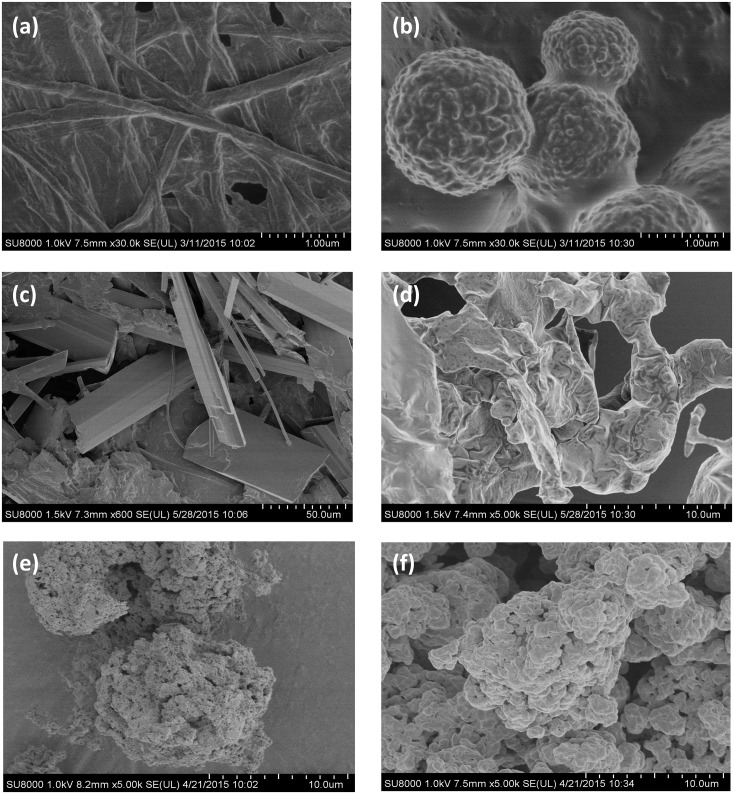
Polyaniline structures observed by SEM. Polymerization was carried out with 7D5L, 15 mM aniline and 5mM of different templates. Nanofibers obtained with SDBS (a). Granular spheres obtained with 5 mM AOT (b). Splinters obtained with 5 mM SDS (c). Amorphous agglomerates obtained with 5 mM SLES (d). Amorphous agglomerates obtained after washing (a) with ethanol (e). Micro-sized granular agglomerates of commercial PANI (f).

Oxidation of aniline by 7D5L in the absence of template gave no structured product due to the irregular branched polymerization of aniline (data not shown). On the contrary, enzymatic polymerization in the presence of SDBS as template led to a nanofiber-structured PANI (Fig 6a). In theory, nanofibers are formed during the initial polymerization stages and they serve as a scaffold for the growth of new PANI particles. If homogeneous nucleation occurs, well-dispersed PANI nanofibers are obtained, whereas heterogeneous nucleation leads to particle aggregation [32]. Similarly, after template's removal, PANI structure was lost and the particles aggregated in micron-sized agglomerates (Fig 6e) due to strong inter-molecular H-bonding between the chains' backbones [33]. Other PANI nano- and micro-scale structures were obtained by varying the doping template: granular spherical particles with AOT, splintered PANI with SDS, and amorphous PANI with SLES (Fig 6b–6d), whereas commercial PANI showed a granular morphology of irregular shaped micron-sized agglomerates (Fig 6f) as described for PANI obtained by chemical synthesis with ammonium peroxydisulfate in a strong acid environment [34].

The uniform size and morphology of the nanofibers obtained with laccase and SDBS provide higher surface area that result in superior performance of the polymer to be readily cast into uniform films. Also, the outstanding water dispersibility of nanofibers facilitates their interaction with ions in solution, enabling nanofibers to be uniformly modified, thus giving rise to superior functionalities in environmentally friendly processing and biological applications [3,32].

On the other hand, PANI size-distribution profiles detected by light-scattering are in agreement with their respective structures observed by SEM. Green PANI synthesized with 7D5L and SDBS showed an hydrodynamic radius ranging between 10 and 290 nm (S4a Fig), whereas the radius varied between 14 and 80 nm in the green PANI synthetized in the presence of AOT (S4b Fig). The remarkably broader size distribution profile of PANI obtained using SDBS as template correlates with its nanofibered structure, whereas the narrow size-distribution profile of PANI obtained with AOT is in concordance with the uniformity of PANI particles (around 1μM-size spheres).

#### Electrochemistry of PANI

**Fig 7:**
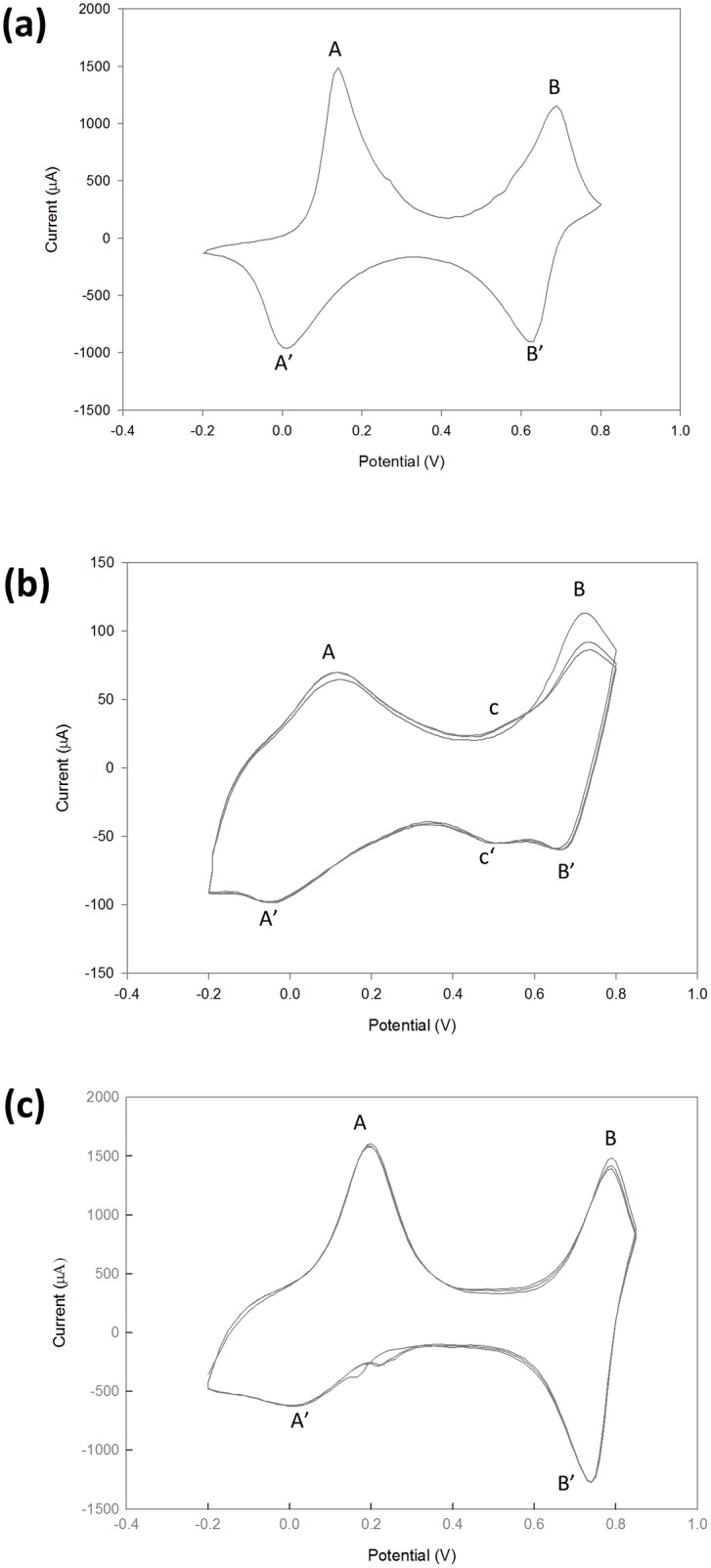
Cyclic voltammetry curves of PANI obtained with 7D5L and SDBS (a) or AOT (b) as templates (after isolation with ethanol) compared with commercial emeraldine salt (c).

The electrochemical responses of green PANI synthetized enzymatically were evaluated by cyclic voltammetry. The voltammetric behaviors of thin films of the green polymers obtained with SDBS or AOT as templates were very similar to that of commercial emeraldine salt (Fig 7). Two redox processes were observed, which correspond to the leucoemeraldine/emeraldine transition (A/A') and emeraldine/pernigraniline (B/B') forms. However, some differences were observed with increasing number of voltammetric cycles. While the response was maintained in the PANI obtained in the presence of SDBS (Fig 7a), a new redox pair (c/c') [9] appeared in the polymer obtained with AOT as template (Fig 7(b)). This is an evidence of higher oxidation stability for the former as the c/c' peaks are attributed to the double-electron redox transition between *p*-benzoquinone and the *p*-hydroquinone through hydrolysis of PANI [35]. We corroborated their origin as degradation products of PANI (by using potentials higher than 0.7 V [36]) when we used a short range potential (from -0.2 V to +0.6 V) and these peaks were missing (data not shown). The redox peaks for PANI synthesized with 7D5L and SDBS were notably sharper and more intense than those for PANI obtained with AOT (at same reaction conditions), most likely due to the polymer’s morphology. In conclusion, the use of SDBS as template resulted in better PANI redox electrochemical properties as regards the rest of anionic surfactants tested (data not shown) or even the chemically synthesized emeraldine salt (Fig 7c).

Finally, we evaluated the conductivity of the green PANI synthesized with 7D5L and SDBS by the four-probe method. The polymer was electro-conductive, with conductivity values around 1.1x10^-5^ S/cm after removing the template with ethanol and water. Since dedoping of the sample occurs during washing, we re-doped the washed sample with 1 M HCl and the polymer's conductivity was rised two orders of magnitude to 2.4x10^-3^ S/cm. Even if studies on enzymatic polymerization of aniline scarcely illustrate the electro-conductivities of the resulting polymers [2,37], this value is three orders of magnitude higher than that reported for PANI obtained after 18 days of reaction with *Trametes versicolor* laccase and AOT (after re-doping with camphor-10-sulfonic acid) [12]. We also use milder reaction conditions (much less enzyme, room temperature, shorter reaction times) than other polymerization reactions catalyzed by laccase [12,38,39]. On the other hand, conductivities of PANI obtained by conventional means range from 10^−10^ to 27 S/cm [40,41], although conductivity values above 10^−3^ S/cm are only attained in strongly acidic media.

Finally, the nanofibered green PANI synthesized here might be very useful as chemical sensor, since uniform nanofibered films respond fast and with high sensitivity to an acid, by becoming more conducting, and the doped form responds to a base by becoming more insulating [33].

## Conclusions

The laccase used here (7D5L) displays outstanding activity on aniline and stability at the conditions required for the synthesis of green polyaniline (emeraldine salt), as compared with other fungal laccases, enabling the use of low amounts of biocatalyst. The systematic characterization of the polymers obtained in the presence of different anionic surfactants revealed the profound effect of the doping template in polyaniline's properties and permitted the adjustment of the reaction conditions to boost the enzymatic synthesis of emeraldine salt. By using a first-class laccase and SDBS as template, we developed an environmentally-friendly method to produce water-soluble conductive PANI with excellent electrochemical properties in up to 75% conversion yield. Its nanofibered structure provides additional advantages like a porous structure and a large surface-to-volume ratio.

## Supporting Information

S1 Fig Structural formulas of the different oxidation and protonation states of polyaniline: Leucoemeraldine, Emeraldine base, Emeraldine salt (bipolaron form) and Pernigraniline. (PDF)

S2 Fig Scheme of the synthesis of polyaniline catalyzed by laccase in the presence of SDBS. (PDF)

S3 Fig MALDI-TOF spectra of enzymatic PANI synthesized with 0.1 U/mL of 7D5 laccase, 15 mM aniline and 0.6 mM AOT (a), 15 mM SDS (b) or 15 mM SDBS (c) compared with commercial Emeraldine salt (d). (PDF)

S4 Fig DLS analysis of PANI synthesized with 7D5 laccase in the presence of 5 mM SDBS (a) and 5 mM AOT (b). (PDF)
