# Supplementary figures and images for: Advanced Synthesis of Conductive Polyaniline Using Laccase as Biocatalyst
Source: PLoS One. 2016 Oct 14;11(10):e0164958. doi: 10.1371/journal.pone.0164958 (PMC5065195; doi:10.1371/journal.pone.0164958)

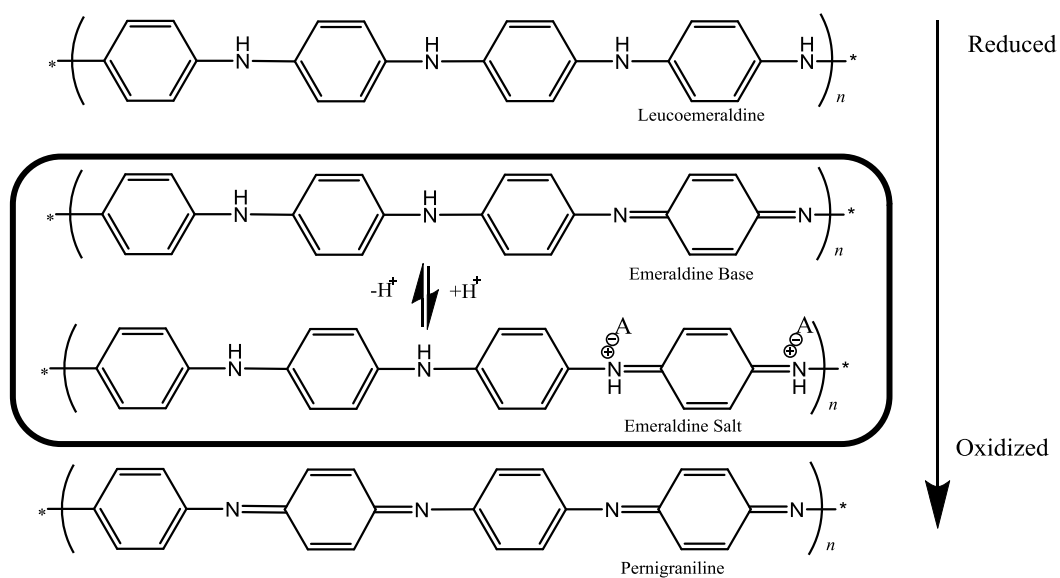

**S1 Fig.** Structural formulas of the different oxidation and protonation states of polyaniline.

Supplement: S1 Fig — (PDF) [file pone.0164958.s001.pdf]

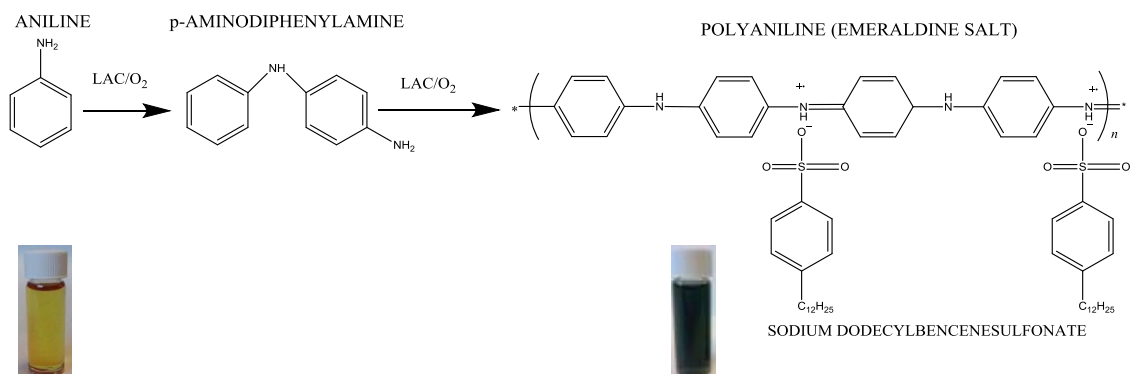

**S2 Fig.** Scheme of the synthesis of polyaniline catalyzed by laccase in the presence of SDBS

Supplement: S2 Fig — (PDF) [file pone.0164958.s002.pdf]

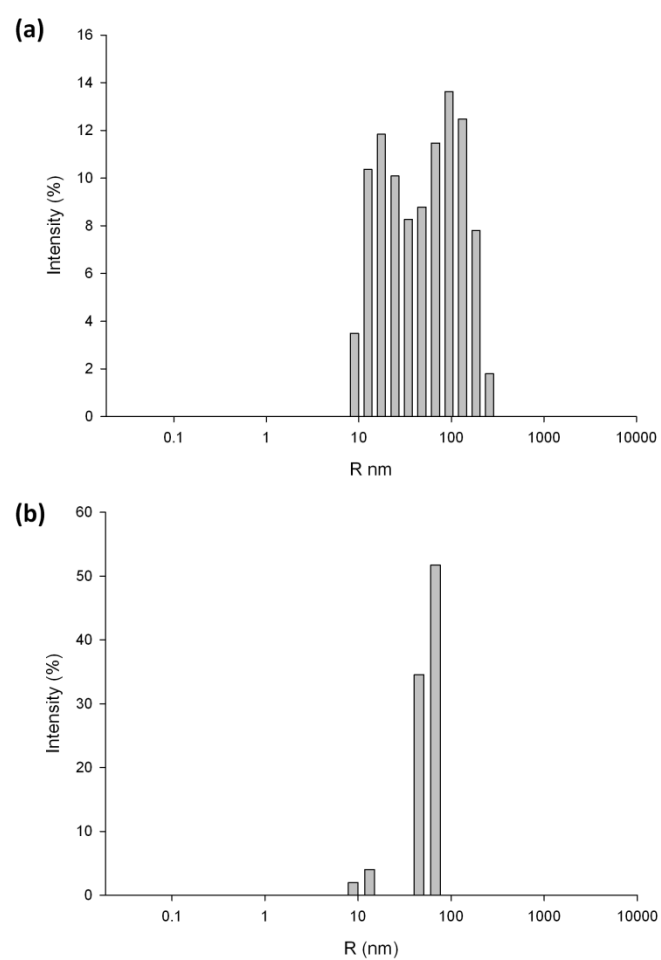

**S4 Fig.** DLS analysis of PANI synthesized with 7D5 laccase in the presence of 5 mM SDBS (a) and 5 mM AOT (b).

Supplement: S4 Fig — (PDF) [file pone.0164958.s004.pdf]
