# Supplementary material for: Advanced Synthesis of Conductive Polyaniline Using Laccase as Biocatalyst
Source: PLoS One. 2016 Oct 14;11(10):e0164958. doi: 10.1371/journal.pone.0164958 (PMC5065195; doi:10.1371/journal.pone.0164958)
Supplement: S3 Fig — (PDF) [file pone.0164958.s003.pdf]

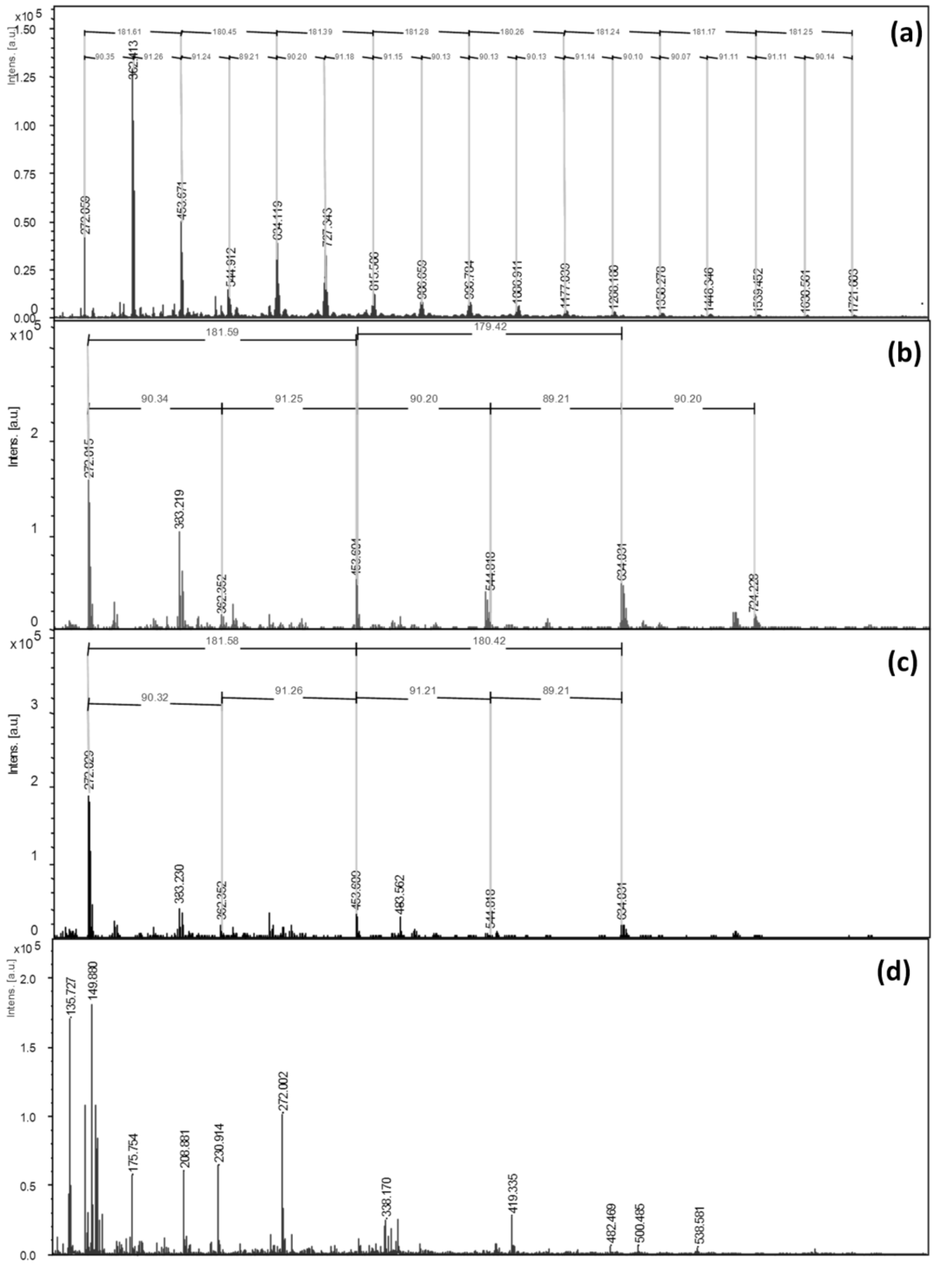


**S3 Fig.** MALDI-TOF spectra of enzymatic PANI synthesized with 0.1 U/ml of 7D5 laccase, 15 mM aniline and 0.6 mM AOT (a), 15 mM SDS (b) or 15 mM SDBS (c) compared with commercial Emeraldine salt (d).
